# Editorial: Application of novel statistical and machine-learning methods to high-dimensional clinical cancer and (multi-) omics data volume II

**DOI:** 10.3389/fgene.2024.1487893

**Published:** 2024-09-25

**Authors:** Chao Xu, Shaolong Cao, Md Ashad Alam

**Affiliations:** ^1^ Department of Biostatistics and Epidemiology, University of Oklahoma Health Sciences Center, Oklahoma, OK, United States; ^2^ Biogen Inc., Cambridge, MA, United States; ^3^ Ochsner Research, Ochsner Clinic Foundation, New Orleans, LA, United States

**Keywords:** multi-omics analysis, high-dimensional data analysis, cancer genomics, prediction analysis, integrative analysis, medical imaging analysis

Three years ago, in 2021, our first Research Topic on Application of Novel Statistical and Machine-learning Methods to High-dimensional Clinical Cancer and (Multi-) Omics Data has been a highlight for the readership in Frontiers, with over 52K views and 13K downloads. It has contributed greatly to the field by highlighting cutting edge research in the area of statistical genetics and methodology. Building on the success of the first volume, we bring another Research Topic of insightful and thought-provoking research on this Research Topic by presenting four articles.

In this second volume, we continue our previous focus on the development and application of novel statistical and machine-learning methods for high-dimensional clinical and (multi-)omics data in cancer-related research. With the development of artificial intelligence (AI), especially the deep learning (DL), three out of four articles in Volume II investigated methods in multi-omics data integration using DL, while the fourth article investigated a new method for sequencing data processing.

With the rapid evolvement of DL, significant progress has been made in applying DL based method to multi-omics integration. In a review article, Wekesa and Kimwele comprehensively discussed the recent trends in using DL techniques for multi-omics data analysis in disease diagnosis, prognosis, and treatment. They focused particularly on multi-omics datasets that involve non-coding RNAs, such as miRNAs and long non-coding RNAs (lncRNAs), which played essential roles in cancer development and research. Several novel DL methods for integration and interpretation were highlighted, including contrastive learning, DeepLIFT, factorization machine deep learning (FMDNN), and graph neural networks (GNNs). Further, they assessed studies combing DL methods and emerging technologies, such as blockchain and internet of things (IoTs), in computational biology. Cases studies in breast and brain cancer detection demonstrated how integrating cutting-edge technologies and DL methods could advance the cancer research and clinical applications. By reading this review, it becomes clear that the development of innovative methods, algorithms, and analytical frameworks that integrate clinical, multi-omics, and imaging data for cancer research is particularly exciting. Moreover, they discussed potential challenges and future prospects, providing valuable insights into the field’s future.

In addition to the data types discussed in Volume I, we aim to showcase more studies that analyze imaging data, particularly due to the extensive use of imaging technique in cancer diagnosis, treatment, and research. Zhao et al. developed new models that can integrate radiomics data and whole genome sequencing data. Although their prediction outcome focused on proximal femoral strength related to hip fracture, their models can be straightforwardly adapted for imaging analysis in cancer research.

Specifically, they extended the DL method of variational autoencoder from a single-view input into a multi-view input approach. Compared to other high-dimension multi-view information integration algorithms, the proposed model demonstrated superior performance in terms of root mean squared error (RMSE) and the coefficient of determination (R-squared). The significance of the analyzed features/variables was further interpreted through the leave-one-out technique.

Another compelling study in this Research Topic explores a linear dimensionality reduction method using DL. Dimension reduction is a critical step in the analysis of high-dimensional genetic and imaging data, as it helps to extract representative features for visualization or downstream analysis, such as prediction or classification. Li et al. introduced neural principal component analysis (nPCA), which enhances the widely-used original principal component analysis (PCA) by retaining the linear information of raw data. This new method was successfully applied to high-dimensional single-cell RNA sequencing datasets of pancreas. The nPCA method holds promise as an alternative dimension reduction technique for cancer investigators.

The last article in this Research Topic addressed the issue of sequencing data compression. With the reduction in sequencing cost, multi-omics data are increasingly generated through sequencing technologies. While bioinformatician and biostatistician often worked with processed sequencing file, such as bam and VCF files, the large raw sequencing files still need to be stored for backup, sharing, and legal requirements. Chen et al. presented a two-step framework for sequencing data compression, achieving up to a four-fold compression ratio compared to Gzip, all within an acceptable timeframe. Their tool, repaq, is freely available on GitHub, providing a valuable solution for managing large-scale sequencing data efficiently.

In summary, the Volume II Research Topic of original research, review, and technology papers highlights the latest advancements in the integrative analysis of clinical, imaging, and (multi-)omics cancer data, along with statistical and computational methods for high-dimensional data analysis. Combined with Volume I, we hope these Research Topic will contribute to the integrative cancer research and inspire further methodology development in related fields.

